# No Support for the Neolithic Plant Invasion Hypothesis: Invasive Species From Eurasia Do Not Perform Better Under Agropastoral Disturbance in Early Life Stages Than Invaders From Other Continents

**DOI:** 10.3389/fpls.2022.801750

**Published:** 2022-02-11

**Authors:** Ginevra Bellini, Alexandra Erfmeier, Karin Schrieber

**Affiliations:** ^1^Department of Geobotany, Institute for Ecosystem Research, Kiel University, Kiel, Germany; ^2^Cluster of Excellence ROOTS, Kiel University, Kiel, Germany

**Keywords:** agropastoralism, biological invasions, disturbance, germination, Neolithic Plant Invasion hypothesis, pre-adaptation

## Abstract

Pre-adaptation to disturbance is an important driver of biological invasions in human-altered ecosystems. Agropastoralism is one of the oldest forms of landscape management. It surged 12,000 years ago in Western Asia and it was then imported to Europe starting 8,000 years ago. The Neolithic Plant Invasion hypothesis suggests that Eurasian plants succeed at invading agroecosystems worldwide thanks to their adaptation to agropastoralism, which derives from these species’ long co-evolution with such practice. Plant species from Western Asia are predicted to have the highest degree of adaptation to agropastoralism, since they have co-evolved with such practice for several millennia more than European plants, and non-Eurasian species should be poorly adapted due to their relatively short exposure. However, this Eurocentric perspective largely ignores that several other cultures around the world independently developed and implemented agropastoralism through history, which challenges this hypothesized superior adaptation of Eurasian species. Here, we tested whether the early-life performance of invasive plants under disturbance depends on their geographical origin and the associated assumed exposure time to agropastoralism. We selected 30 species divided into three groups: exposure long, native to Western Asia; exposure medium, native to Central Europe; exposure short, native to America. Three soil disturbance treatments (control/compaction/tilling) combined with two space occupancy levels (available/occupied) were applied to monospecific experimental units (*n* = 900), each containing 50 seeds. We predicted that Eurasian species would benefit more from disturbance in terms of germination and seedling performance than species with shorter assumed exposure to agropastoralism, and that this effect would be stronger when space is occupied. Contrary to these expectations, all species groups profited equally from disturbance, while non-Eurasian species were most hampered by space occupancy. For germination success and speed, exposure long species had higher values than exposure short species, regardless of the disturbance treatment. These results do not support that Eurasian species possess a higher adaptation to agropastoralism, but rather that non-Eurasian species can cope just as well with the associated disturbances. We discuss how future experiments that address the complex relationships between species interactions, plant life-phases and the quality of disturbance can help to understand the role of land-use history in plant invasion success.

## Introduction

Identifying the mechanisms that promote or impede biological invasions remains a challenging goal in plant ecology and evolution research. In recent years, much attention has been paid on the role of species pre-adaptation, particularly for invasions in human-impacted landscapes (Bayliss et al., 2017; Rosche et al., 2018). In fact, human intervention has shaped landscapes on Earth for thousands of years (Ellis et al., 2021), and successful herbaceous plant invaders thus tend towards a more ruderal strategy (*sensu* Grime, 1977) and may have adapted to human-dominated (highly disturbed) environments in their native range (Guo et al., 2018). It has been suggested that agropastoral disturbance and pre-adaptation to such practice are the two key drivers behind the success of Eurasian plants as invaders of agropastoral ecosystems worldwide [Kalusová et al., 2017; Monnet et al., 2020; Neolithic Plant Invasion (NPI) hypothesis, MacDougall et al., 2018]. According to the NPI hypothesis, most plant species outside of Eurasia lack this kind of pre-adaptations as they have experienced agropastoralism only after the first contact with European settlers starting in the 15th century. However, the NPI hypothesis disregards the existence of ancient agropastoral practices in several continents besides Eurasia, and that consequently adaptation of local native plants to such practice might have evolved in parallel at various locations (Clement et al., 2015; Hilbert et al., 2017; Chomicki et al., 2020). In this study, we present the outcomes of a multi-species experiment investigating whether plant invaders from Eurasia indeed perform better under agropastoral disturbance than plant invaders from other regions of the world, as predicted by the NPI hypothesis.

Processes associated with successful invasions display great variability when studied across taxa and contexts (Kueffer et al., 2013). Nevertheless, numerous empirically supported hypotheses highlight the importance of particular species traits such as high reproduction rates, germination success and competitive ability. Such traits can be developed through the individual or combined effects of evolution following introduction (post-adaptation) and fortunate similarity between old and new habitat (pre-adaptation) (Guo et al., 2014). Since human-disturbed habitats seem particularly prone to invasion (Jauni et al., 2015), increasing attention has been posed to pre-adaptation to anthropogenic disturbance and its link to invasions (Hufbauer et al., 2012; Kalusová et al., 2021). Disturbance considerably shifts local abiotic and biotic conditions and consequently resource availability (Pearson et al., 2018), thereby affecting habitat structure and ecosystem function. Plant species that have evolved in disturbance-prone environments can tolerate rapid environmental changes and quickly exploit released resources (Kawecki, 2000; Lee and Gelembiuk, 2008), which is likely a great advantage during an invasion process. Tolerance to anthropogenic disturbance is particularly essential in early life stages including germination and seedling establishment (Hofmann and Isselstein, 2003), which are delicate yet critical for a successful invasion (Hantsch et al., 2013; Hock et al., 2015; Gioria and Pyšek, 2017).

Pre-adaptation to disturbances associated with agropastoral management may be of particular importance when considering that nowadays about half of the planet’s inhabitable surface is dedicated to livestock rearing (77%) and agriculture (23%) (FAO, 2021), which is leading to profound landscape transformations in many countries (Carvalho et al., 2020). In addition, such areas are particularly impacted by the ever-increasing exchange of plant species worldwide. The European continent has been one of the hubs of economic and cultural exchange for centuries and has consequently experienced both species introduction and exportation (van Kleunen et al., 2015; Seebens et al., 2017). Numerous European plants that have been introduced worldwide – starting from the 15th century – are now successful invaders of agropastoral ecosystems (La Sorte and Pyšek, 2009; Kalusová et al., 2017). According to the NPI hypothesis (MacDougall et al., 2018), Eurasian plants are particularly successful at invading these ecosystems in other continents because of their assumed pre-adaptation to agropastoralism, a disturbance to which most plants outside Eurasia are poorly adapted. Such agropastoral practices arose around 10,000 BCE in Western Asia from where they were introduced around 6,000 BCE to Europe (Hejcman et al., 2013; Shennan, 2018). In the Neolithic, the introduction of agropastoralism involved an extensive anthropogenic landscape transformation of the European continent. Large stands of forest were cleared over a few millennia in favor of crop fields and pastures, which were exposed to novel anthropogenic disturbance regimes. Crop cultivation required tilling and plowing, which disrupted soil surface layers with tools pulled by domesticated animals. Such practices cause perturbation to root systems and associated microbiota, create open soil patches, and foster the vertical migration of seeds into the soil (Colbach et al., 2014). Livestock rearing is another practice connected to agropastoralism, which affected local vegetation in Neolithic Europe. The enclosure of livestock lead to severe soil compaction and mechanical vegetation damage via trampling, increased nutrient input through excretes, as well as intense and frequent biomass removal via grazing (Cingolani et al., 2003; Drewry et al., 2008). Along with these practices, many plant species – referred to as Archaeophytes – were introduced from Western Asia to Europe during the Neolithic (Lambdon et al., 2008). Archaeophytes had co-existed with agropastoralism for millennia before being introduced to Europe, and thus they possessed pre-adaptations that likely allowed them to endure the associated disturbances and thrive in their new range (Europe) (MacDougall et al., 2018). The local European species persisting under this severe landscape transformation either adapted to the new anthropogenic disturbance or shifted in their realized niches (Eriksson, 2013). Known adaptations to agropastoral management are, for example, the production of numerous small seeds with high persistence in the soil seed bank (Albrecht and Auerswald, 2009), the ability to germinate rapidly upon the creation of open soil patches, the capacity to successfully germinate in highly compacted/deep soil layers (Place et al., 2008) and compensatory growth following biomass removal. After the 15th century CE, Archaeophytes and disturbance-adapted European species were exported with the agropastoral practices to other continents (Crosby, 1986). In these new regions, they thrived and often outcompeted local plants, that are assumed to have had only little to no contact with agropastoralism until then, according to the NPI hypothesis.

However, the NPI hypothesis does not fully take into consideration that Eurasia is neither the only nor the first region to have implemented agropastoralism on a large scale. Such practices emerged independently in several other areas, such as eastern North America (2,500–2,000 BCE), sub-Saharan West Africa (∼ 2,000 BCE), and the Indian sub-continent (3,000–2,000 BCE) (Purugganan and Fuller, 2009). It is therefore questionable whether plant species from outside of Europe have indeed not been in intensive contact with agropastoral disturbance until the arrival of European settlers starting from the 15th century CE. The emergence and broad implementation of agricultural practice in these areas is evinced by the appearance of crop weeds in archaeobotanical remains (Vigueira et al., 2013). In some forested regions of North America, for example, after the introduction of maize cultivation around 500 CE, archaeobotanical remains exhibited higher species diversities as they suddenly included various grasses, legumes and weeds such as *Ambrosia trifida* L. and *Polygonum* spp. (Asch Sidell, 2007). In this particular region, the transition from hunting and gathering to agropastoralism prior to the arrival of European settlers was gradual and the practices coexisted for about 4,000 years, which has likely enabled local species to slowly adapt to novel disturbance (Lemmen, 2013). The fact that species native to such regions, e.g., *Galinsoga parviflora* Cav. are even a nuisance in agropastoral ecosystems in Eurasia (Warwick and Sweet, 1983) support this assumption. Finally, for the particular case of livestock-associated disturbance, one cannot ignore that many regions of the world harbored large native herbivores (bison, deer, vicuñas, etc.) which have maintained species-rich pastoral ecosystems through grazing prior to the introduction of pastoralism by humans (Mueller et al., 2020). In summary, it is highly likely that many non-Eurasian species have adapted to cropping practice, grazing and trampling prior to the “introduction” of the agropastoral lifestyle from Eurasia.

We test the NPI hypothesis by following the premise that invasive plants originating from Western Asia have been exposed for the longest time to agropastoralism, followed by species originating in Europe and then by (other) non-Eurasian species. We used a multi-species experiment involving 30 species that are invasive in agropastoral ecosystems outside their native range by comparing their germination and seedling establishment success under different disturbance regimes associated with agropastoral management. Following MacDougall et al. (2018), our plant species fall into three groups according to their native distribution range and assumed associated historical exposure to agropastoralism: EL – exposure long, native to Western Asia; EM – exposure medium, native to Central Europe; ES – exposure short, native to America. Each individual species was exposed to three types of disturbance (none/soil compaction/soil surface tilling) and two levels of soil space occupancy (full space available/space occupied with the grass *Festuca rubr*a L.) in a fully factorial experimental design. We selected *F. rubra* as a grass species to serve as a common ground cover, since the species has a broad native distribution range spanning Eurasia and America (Canadensys, 2021; GBIF, 2021) and thus shares co-evolutionary history with the vast majority of our species. In accordance with the NPI hypothesis, we expect that (i) disturbance and space availability without competitors would benefit early-life performance of all species; (ii) the positive effect of disturbance increases with assumed length of exposure to agropastoralism (EL species performing best, then EM and then ES species); (iii) the positive effect of space availability increases with time of exposure to agropastoralism (EL species performing best, then EM and then ES species), and these differences will be magnified by disturbance.

## Materials and Methods

### Study Species and Seed Material

Among all possible plant species that have successfully invaded agropastoral ecosystems outside their native distribution range, we selected 30 species belonging to three groups: EL (exposure long, native to Western Asia, represented by nine species), EM (exposure medium, native to Europe, represented by eleven species), and ES (exposure short, native to America, represented by ten species) (Table 1). The study species belong to the life forms of cryptophytes, therophytes, geophytes, or hemicryptophytes (Raunkiær, 1907), as assessed through the TRY plant trait database (Kattge et al., 2020). Exposure-long species (EL) have an Archaeophyte status in the majority of European countries (Preston et al., 2004; Klotz et al., 2021), and have been reported to be invasive outside of Europe (Swearingen and Bargeron, 2016; CABI, 2020; ISSG, 2021). Exposure-medium species (EM) are native to Europe (Bundesamtes für Naturschutz, 2003; Kalusová et al., 2017; Klotz et al., 2021) and are invasive in agropastoral ecosystems worldwide (ISSG, 2021; Swearingen and Bargeron, 2016; CABI, 2020). Exposure-short species (ES) are plants that have invaded Europe’s agropastoral ecosystems and have been introduced from the American continent after the 15th century CE (Kattge et al., 2020).

**TABLE 1:** Overview of species composition, seed material accessions and seed pre-treatments of the three target groups of plants: EL, EM, and ES, respectively, long-, medium-, and short- assumed exposure to agropastoralism.

Exposure- length group	Scientific name	Accessions	Geographic origins Country code – City (Number sources)	Collection year	Dormancy-breaking treatment	Habitat in native range	Habitat in invaded range
EL	*Agrostemma githago* L.	4	DE-Bonn (1) DE-Darmstadt (2) DE-Wasbek (1)	2019 2018, 2018 2020	W	Cultivated areas (Klotz et al., 2021)	Cultivated areas, disturbed areas (Invasive.org, 2018)
EL	*Anchusa officinalis* L.	5	AT-Vienna (1) DE-Berlin (1) DE-Bonn (1) DE-Konstanz (1) DE-VWW UG 1 (1)	2017 2019 2018 2016 2019	/	Meadows, urban areas (Klotz et al., 2021)	Grasslands, roadsides, pastures (Fraser Valley Invasive Species Society, 2021)
EL	*Cichorium intybus* L.	7	DE-Berlin (1) DE-Bonn (1) DE-Darmstadt (1) DE-Halle (Saale) (1) DE-VWW UG 1 (1) DE-VWW UG3 (1) KS-Košice (1)	2018 2019 2019 2019 2019 2018 2019	/	Meadows, urban areas (Klotz et al., 2021)	Cultivated areas, disturbed areas, roadsides (Swearingen and Bargeron, 2016)
EL	*Cyanus segetum* Hill	3	DE-Darmstadt (2) DE-Göttingen (1)	2018, 2018 2019	/	Cultivated areas, meadows (Klotz et al., 2021)	Cultivated areas, grasslands, meadows (Invasive Species Council of British Columbia, 2021)
EL	*Dipsacus fullonum* L.	3	DE-Bonn (1) DE-Darmstadt (1) DE-Konstanz (1)	2019 2019 2018	/	Meadows, riverbanks, urban areas (Klotz et al., 2021)	Cultivated areas, grasslands, meadows (CABI, 2020)
EL	*Papaver rhoeas* L.	5	DE-Bonn (1) DE-Darmstadt (2) DE-Wasbek (1) DE-Leipzig (1)	2019 2018, 2019 2020 2019	C	Cultivated areas, meadows, perennial heaps (Klotz et al., 2021)	Cultivated areas (CABI, 2020)
EL	*Thlaspi arvense* L.	3	DE-Darmstadt (2) DE-Halle (Saale) (1)	2018, 2018 2019	W	Cultivated areas, meadows (Klotz et al., 2021)	Cultivated areas (Koop, 2018)
EL	*Tripleurospermum inodorum* (L.) Sch.Bip.	1	DE-Berlin (1)	2019	/	Meadows (Klotz et al., 2021)	Cultivated areas (Koop, 2018)
EL	*Vicia sativa* L.	1	DE-Bruno Nebelung GmbH (1)	2020	/	Cultivated areas (Klotz et al., 2021)	Cultivated areas (Koop, 2018)
EM	*Capsella bursa-pastoris* (L.) Medik.	4	DE-Bonn (1) DE-Darmstadt (1) DE-Kiel (2)	2019 2018 2020, 2020	W	Cultivated areas, meadows, pastures, urban areas (Klotz et al., 2021)	Cultivated areas (CABI, 2020)
EM	*Carum carvi* L.	4	DE-Bonn (1) DE-Darmstadt (2) DE-Halle (Saale) (1)	2018 2018, 2019 2018	C	Meadows, pastures (Klotz et al., 2021)	Forest openings, meadows (Alberta Invasive Species Council, 2021)
EM	*Cirsium vulgare* (Savi) Ten.	3	DE-Kiel (3)	2020, 2020, 2020	W	Forest clearings, meadows, urban areas (Klotz et al., 2021)	Cultivated areas, rangelands, riverbanks, roadsides (CABI, 2020)
EM	*Cynoglossum officinale* L.	4	DE-Darmstadt (1) DE-Göttingen (1) DE-VWW UG 5 (1) DE-VWW UG22 (1)	2019 2019 2020 2020	S+C	Meadows, urban areas (Klotz et al., 2021)	Cultivated areas, pastures, rangelands, roadsides (CABI, 2020)
EM	*Daucus carota* L.	4	DE-Bonn (1) DE-Darmstadt (1) DE-Leipzig (1) DE-Konstanz (1)	2019 2019 2018 2018	C	Grasslands, meadows, urban areas (Klotz et al., 2021)	Disturbed areas, grasslands, meadows (CABI, 2020)
EM	*Hypochaeris radicata* L.	6	AT-Bad Kleinkirchheim (1) DE-Bonn (1) DE-Kiel (2) DE-VWW UG1 (1) DE-VWW UG22 (1)	2016 2019 2020, 2020 2019 2019	C	Meadows, pastures (Klotz et al., 2021)	Cultivated areas, grasslands, meadows, urban areas (CABI, 2020)
EM	*Linaria vulgaris* Mill.	4	DE-Bonn (1) DE-Darmstadt (1) DE-VWW UG1 (1) DE-VWW UG5 (1)	2019 2018 2019 2019	C	Cultivated areas, forest clearings, meadows, urban areas (Klotz et al., 2021)	Abandoned cultivated areas, pastures, rangelands, riparian corridors, roadsides (ISSG, 2021)
EM	*Rumex acetosella* L.	4	DE-Bonn (1) DE-Darmstadt (2)	2018 2017	W	Cultivated areas, forest clearings, meadows (Klotz et al., 2021)	Cultivated areas, meadows, lawns, roadsides (ISSG, 2021)
EM	*Silene vulgaris* (Moench) Garcke	6	AT-St. Lorenzen (1) DE-Bonn (1) DE-Darmstadt (1) DE-Kiel (1) DE-Leipzig (1) DE-Göttingen (1)	2019 2019 2019 2020 2019 2018	/	Grasslands, meadows, urban areas (Klotz et al., 2021)	Cultivated areas, disturbed areas, roadsides (Minnesota Wildflowers, 2021)
EM	*Sonchus asper* (L.) Hill	5	DE-Kiel (4) DE-Wasbek (1)	2020, 2020, 2020, 2020 2020	/	Cultivated areas, meadows, urban areas (Klotz et al., 2021)	Cultivated areas, disturbed areas, pastures, roadsides (ISSG, 2021)
EM	*Trifolium pratense* L.	1	DE-Bruno Nebelung GmbH (1)	2020	/	Lawns, meadows, pastures (Klotz et al., 2021)	Forest clearings, meadows (White, 2013)
ES	*Claytonia perfoliata* Donn ex Willd.	2	DE-Bonn (1) DE-Darmstadt (1)	2019 2019	C	Mountain meadows, grasslands, sagebrush (Matthews, 1993)	Gardens, lawns (Ries et al., 2021)
ES	*Erigeron annuus* (L.) Pers.	3	DE-Berlin (1) DE-Halle (Saale) (1) DE-Konstanz (1)	2019 2018 2019	/	Abandoned areas, cultivated areas, roadsides (Lady Bird Johnson Wildflower Center, 2017)	Disturbed areas, forests, grasslands (Pacanoski, 2017)
ES	*Erigeron canadensis* L.	4	DE-Konstanz (1) DE-Kiel (2) DE-Wasbek (1)	2018 2020, 2020 2020	/	Cultivated areas, grasslands, riparian areas (Tilley, 2012)	Cultivated areas, grasslands (Swearingen and Bargeron, 2016)
ES	*Galinsoga parviflora* Cav.	4	DE-Berlin (1) DE-Halle (Saale) (1) DE-Kiel (1) DE-Wasbek (1)	2019 2018 2020 2020	/	Cultivated areas, disturbed areas, gardens, grasslands (Rzedowski and Calderón de Rzedowski, 2008)	Cultivated areas, urban areas (Damalas, 2008)
ES	*Lupinus polyphyllus* Lindl.	4	DE-Kiel (2) DE-Konstanz (1) DE-Probsteierhagen (1)	2020, 2020 2019 2020	/	Meadows, riverbanks, roadsides (Fremstad, 2010)	Disturbed areas, gardens, roadsides (Fremstad, 2010)
ES	*Matricaria discoidea* DC.	5	DE-Darmstadt (1) DE-Halle (Saale) (1) DE-Kiel (1) DE-Wasbek (1)	2019 2018 2020 2020	/	Ruderal areas (Flora of North America, 2021)	Disturbed areas, pastures, urban areas (Flora of North America, 2021)
ES	*Oenothera glazioviana* Micheli	1	DE-Darmstadt (1)	2019	/	Fallow fields, gardens, railroad tracks, roadsides (Missouri Botanical Garden, 2021)	Open disturbed areas (Flora Digital de Portugal, 2014)
ES	*Oxalis corniculata* L.	2	DE-Wasbek (1) DE-Kolkwitz (1)	2020 2020	/	Cultivated areas, gardens, urban areas (University of California Integrated Pest Management, 2021)	Cultivated areas, gardens, lawns, pastures (ISSG, 2021)
ES	*Phacelia tanacetifolia* Benth.	2	DE-Darmstadt (2)	2019, 2019	/	Open ecosystems in chaparral, sandy slopes, forests below 2,200 m (Smither-Kopperl, 2018)	Disturbed areas, roadsides (Manual of the Alien Plants of Belgium, 2017)
ES	*Solidago canadensis* L.	3	DE-Halle (Saale) (1) DE-Leipzig (1) DE-Konstanz (1)	2018 2019 2019	/	Abandoned pastures and fields, grasslands, forest edges, roadsides, urban areas (Walck et al., 1999)	Abandoned pastures and fields, grasslands, forest edges, roadsides, urban areas (CABI, 2020)

*We also report the species’ habitat in their native and introduced range. Dormancy breaking treatments: C – cold: seeds were placed in darkness at 5°C for 20 days; W – warm seeds were placed in darkness at 25°C for 15 days; S – scarification: the external seed coating was manually removed. Information on dormancy breaking treatments obtained from information available on Baskin and Baskin (2014) and the Royal Botanic Gardens Kew (2021). EL species are native to Western Asia, EM species are native to Europe and ES species are native to America. Plant species taxonomy obtained from WFO (2021).*

We collected the seed material for all three exposure-length groups in Central and Northern European wild populations, as (i) we assume that the pre-adaptations to agropastoral disturbance that support a species’ introduction, establishment and spread in a novel habitat will be detectable in its native as well as in its invaded distribution range; and (ii) to select populations that are adapted to the climate present in our common garden. The final composition of the groups (Table 1) was driven by our intention of having similar plant families and life forms across groups but also by limitations in the availability of the seed material. Seeds were sampled in wild populations in the field or they were provided by botanical gardens (exclusively wild collections) or regional wild seeds companies. For each species, we gathered seed material from as many accessions as possible. For a detailed list of accessions see Table 1. We assigned an equal proportion of seeds from each of the available accessions to each experimental unit in order to avoid that genetic differentiation and maternal effects could confound the effects of out treatments (see next section for details).

### Experimental Setup

To investigate how species with different (assumed) histories of exposure to agropastoralism respond in their early life phases to the combined effects of space occupancy and disturbance, we set up a common garden experiment at the campus of Kiel University (54°20′ N, 10°06′ E) in the summer of 2020. Our experimental units consisted of 900 plastic pots with a volume of 7.5 L each (diameter: 25.5 cm, height: 21 cm) filled with a mixture of 50% fine sand, 35% compost, and 15% clay, which resembles the composition of grassland soils that can be found in Germany (LLUR SH, 2021).

To address the effect of space occupancy on the emergence of the target species, we applied two treatments (space available: SA; space occupied: SO), each to 450 experimental units. Experimental units with occupied space were planted with individuals of the grass species *Festuca rubra* L. (cultivar Dipper, originated in Germany; OECD, 2021). We selected this species based on its wide native distribution range, comprising the whole temperate-cold regions of Eurasia and America, as we aimed at avoiding that particular exposure-length groups would be particularly affected by *F. rubra* because of differences in co-evolution with this species. The *F. rubra* cultivar Dipper is not reported to secrete allelopathic chemicals and was therefore preferably selected. The *F. rubra* individuals used for planting were previously germinated and raised under common garden conditions in flowering beds filled with the above-described soil mix. We added 11 young *F. rubra* individuals in a fixed spatial scheme that ensured an equal surface availability within each experimental unit (Supplementary Material 1). After the grasses had reached a sufficient size (4 weeks after transplanting; height ∼ 7 cm, diameter ∼ 3 cm), we added 50 seeds of either of our 30 study species to each pot, in order to have monospecific experimental units. All species that required a dormancy-breaking treatment were treated accordingly, following information available in the standard reference Baskin and Baskin (2014) and the Seed Information Database of the Royal Botanic Gardens Kew (2021; Table 1). The pre-treated seeds were mixed with 50 mL of fine sand to enable an even distribution on the soil surface. This mixture contained an equal proportion of seeds for all accessions available for a given species. All experimental units were carefully watered with sprinklers immediately after sowing.

We applied three disturbance treatments (no disturbance: DN; compaction: DC; tilling: DT), each to 300 experimental units (ten per treatment per species, half with occupied and half with available space) one day after sowing the seeds into the experimental units. The compaction treatment aimed at reproducing the impact of a cow hoof on the soil. For this purpose, we created a wooden cow hoof (10 cm × 12 cm × 2 cm; impact area ∼100 cm^2^) and placed it at the bottom of a metal rod that served as a soil compactor with a weight of 8 kg. The pressure exerted by a cow hoof step can vary between 130 and 250 kPa (adult Friesian cow – *Bos taurus taurus* L.) (Di et al., 2001). We applied a pressure of 200 kPa by placing the compactor on the soil and adding the weight of a 62 kg person, who stepped on the device three times. To cover the whole surface of the soil, we subdivided the surface area of the experimental unit into four sectors and placed a hoof print as described above in each of them (Supplementary Material 1). The soil tilling treatment aimed at perturbing the topsoil. We employed a three-tined rake to trace lines that covered the entire soil surface. The rake was pulled through the soil of each experimental unit three times in the same direction with a penetration depth of 5 cm.

**FIGURE 1:**
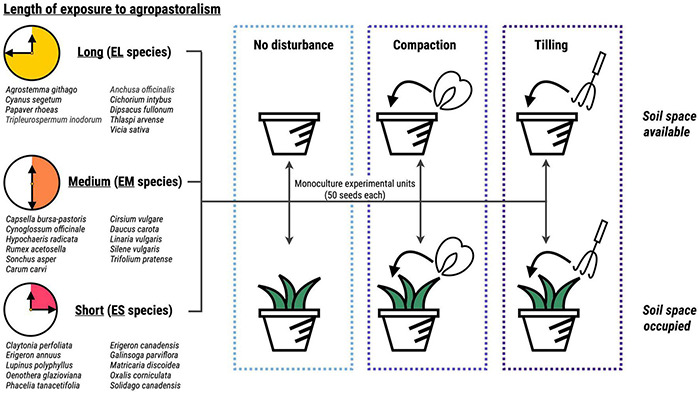
Schematic representation of the experimental setup. We selected species attributable to three groups according to their geographical origin and therefore assumed length of exposure to agropastoralism: yellow – long exposure, orange – medium exposure, red – short exposure. Fifty seeds from a single species were sown into experimental units, which were subject to one of three disturbance treatments (light blue – no disturbance, blue – compaction, purple – tilling), crossed with two levels of space occupancy (white – available, green – occupied). Each species × disturbance × space occupancy combination was replicated with five experimental units. N experimental units = 900.

In summary, our experimental setup comprised 30 species divided in three groups (EL, EM, ES) × 2 space occupancy treatments (SA, SO) × 3 disturbance treatments (DN, DT, DC) × 5 replicates = 900 experimental units containing each 50 seeds (Figure 1). The experimental units were placed on a paved area in 25 rows of 36 elements with a NW-SE orientation and watered with sprinklers when necessary. They were covered with a fine, transparent mesh (0.5 cm openings) to prevent the removal of seeds by animals and minimize the input of seeds from the surrounding environment.

### Data Acquisition

We acquired performance data for two different early life phases, namely the germination and seedling stage. We assessed germination performance by counting the number of germinated individuals per pot twice to thrice per week for a period of 8 weeks. We then calculated germination success $\left(\frac{n_{max}}{n}\right)$, speed $\left(\frac{\sum_{i=1}^{k}n_{i}}{\sum_{i=1}^{k}n_{i}t_{i}}\right)$, and synchronization index $\left(\sum \frac{n_{i}\left(n_{i}-1\right)/2}{\sum n_{i}\left(\sum n_{i}-1\right)/2}\right)$ where *n* is the total number of seeds added (50), n_*max*_ is the total number of seeds germinated, *n*_*i*_ is the number of seeds germinated on the *i*^th^ occasion, *t*_*i*_ is the number of days from sowing to the *i*^th^ observation, and *k* is the last day of germination assessment (Ranal and Santana, 2006). Regarding seedling performance, we calculated seedling survival on the experimental unit level as the proportion of seedlings survived until the end of the experiment out of the total of seeds that germinated. At the end of the experiment, we harvested the total aboveground biomass of all target species individuals within one experimental unit. We did not harvest *F. rubra*. The target species biomass was then dried at 100°C for 24 h. We calculated average target species individual biomass per experimental unit by dividing the cumulative dry biomass by the number of seedlings which survived until the end of the experiment. We can exclude potentially confounding correlations between seed size and survival in our experimental setup. Seed weight, which is a strong predictor for seedling size had a significant positive effect and no negative effect on survival rates in our experiment. In addition, there was no significant difference between the groups in terms of seed mass [Kruskal–Wallis χ^2^_(DF = 2)_ = 3.951, *p* = 0.139, Supplementary Material 2].

### Statistical Analyses

All statistical analyses were performed in R (v4.0.3, R Core Team, 2019) with (generalized) linear mixed-effects models using the R-package glmmTMB (v1.1.3, Brooks et al., 2017). All models included exposure-length group (factor: EL vs. EM vs. ES), space occupancy (factor: SA vs. SO), disturbance (factor: DN vs. DC vs. DT), and all of their interactions as predictors as well as species (factor: 30 levels corresponding to species identity) as a random effect. Initially, we weighted the species random effect by a matrix of exact phylogenetic relatedness estimates among species as determined with the R-package V.PhyloMaker (Jin and Hong, 2019), based on two recently published phylogenetic trees (Qian and Jin, 2016; Smith and Brown, 2018). A custom script to obtain the matrix and integrate it into a glmmTMB modeling framework was obtained from Li and Bolker (2021) (R-package phyloglmm v0.1.0.9001, 2021). However, the resulting models exhibited dispersion and distribution problems that could not be solved with adjustments of error families, link functions, zero-inflation and dispersion formulas or response data transformations (Supplementary Material 2). The same applied for models that included species nested within family as random factor. As these issues likely resulted from unbalanced representation of plant families in our experimental setup, we therefore decided to neglect relatedness effects above and below the species level and maintained the models that included a random effect for species only.

All of our models were fitted with a maximum likelihood approach. We validated them based on residual diagnostic plots and tests provided in the R-package DHARMa (v0.3.3.0, Hartig, 2021). A detailed overview of the chosen distribution families, links and transformations for each model is reported in Table 2. Sum-to-zero contrasts were set on all factors for the calculation of type III ANOVA tables based on Wald-χ^2^ tests (R-package: car v3.0-10, Fox and Weisberg, 2018). In the case of significant interactions between group, disturbance and space occupancy, we calculated *post hoc* contrasts on the estimated marginal means among levels of a given factor only within levels of other factors involved in the respective interaction (R-package: emmeans v1.5.2-1, Lenth, 2021). Variance components were extracted from all models using the R-package insight (v0.14.4, Lüdecke et al., 2019).

**TABLE 2:** Overview of the structure and the results from the Generalized Linear Mixed Effects Models for early life performance responses.

		Germination success	Germination speed	Synchronization index	Seedling survival	Average seedling biomass
Response families and transformations	Error family	Betabinomial	Gaussian	Gaussian	Betabinomial	Gaussian
	Link-function	Logit	Identity	Identity	Logit	Identity
	Response transformation	None	Log	Logit, then scaled with species as grouping factor	None	Log

Fixed effects	Exposure length (EL, EM, ES)	*	***	n.s.	n.s.	*
	Disturbance (DN, DC, DT)	***	•	*	**	n.s.
	Space occupancy (SA, SO)	n.s.	***	•	n.s.	***
	Exposure length × Disturbance	n.s.	n.s.	*	n.s.	n.s.
	Exposure length × Space occupancy	n.s.	n.s.	n.s.	*	***
	Disturbance × Space occupancy	n.s.	n.s.	n.s.	•	n.s.
Exposure	length × Disturbance × Space occupancy	n.s.	n.s.	n.s.	n.s.	n.s.
% Variance explained by	Fixed effects	4.6	16.3	3.4	3.5	35.2
	Random effects (Species)	12.2	16.6	0	8.8	28.8
	Residuals	83.2	67.1	96.6	87.7	36.0

The table provides information on the error families, link functions and transformations used for each of the response variables. Moreover, it shows levels of significance for each fixed effect term as obtained from type III ANOVA based on Wald-χ^2^ tests as well as variance components for each model. EL, exposure long species; EM, exposure medium species; ES, exposure short species; DN, no disturbance; DC, disturbance compaction; DT, disturbance trampling; SA, space available; SO, space occupied. Significance levels: ns: non significant; • : 0.07 p < 0.05; *p ≤ 0.05; **p ≤ 0.01; ***p ≤ 0.001.

## Results

### Exposure Length, Disturbance, and Space Occupancy Main Effects

**FIGURE 2:**
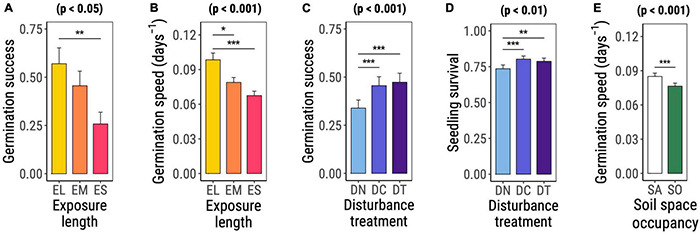
Significant main effects of exposure-length group **(A,B)**, disturbance treatment **(C,D)**, and space occupancy treatment **(E)** on early life performance of the studied invasive plant species as determined based on Wald χ^2^ tests on (generalized) linear mixed effects models (significance level noted on top of the plot). Graphs show the marginal means and standard errors estimated by the corresponding model. Horizontal bars with asterisks highlight significant differences among factor levels assessed based on *post hoc* comparisons on marginal estimated means. Abbreviations and color codes: yellow EL: exposure long species; orange EM: exposure medium species; red ES: exposure short species; light blue DN: no disturbance; blue DC: disturbance compaction; purple DT: disturbance tilling; white SA: space available; green SO: space occupied. Significance levels: *p ≤ 0.05; **p ≤0.01; ***p ≤ 0.001.

Both germination success [χ^2^_(DF = 2)_ = 8.752, *p* = 0.013, Table 2 and Figure 2A] and germination speed [χ^2^_(DF = 2)_ = 21.399, *p* < 0.001, Table 2 and Figure 2B] differed significantly among species groups. For both response variables, species with long exposure (EL) presented the highest values, followed by species with medium (EM) and short (ES) exposure. EL species significantly differed from ES plants in germination success (*t*-ratio_EL–ES_ = 3.010 with *p* = 0.008) and from both EM and ES species in germination speed (*t*-ratio_EL–EM_ = 2.676 with *p* = 0.021, *t*-ratio_EL–ES_ = 4.409 with *p* < 0.001).

Disturbance significantly favored germination success [χ^2^_(DF = 2)_ = 122.332, *p* < 0.001, Table 2 and Figure 2C] and seedling survival [χ^2^_(DF = 2)_ = 10.633, *p* = 0.005, Table 2 and Figure 2D]. Germination success was equally high under soil tilling (DT) and soil compaction (DC) but significantly lower in the control treatment (DN) (*t*-ratio_DN–DC_ = −7.629 with *p* < 0.001, *t*-ratio_DN–DT_ = −8.776 with *p* < 0.001, Figure 2C). In comparison with the control treatment, the application of any disturbance treatment significantly increased seedling survival (*t*-ratio_DN–DC_ = −3.831 with *p* < 0.001, *t*-ratio_DN–DT_ = −2.945 with *p* = 0.009, Figure 2D).

Mean germination speed was the only early life performance trait that was significantly affected by space occupancy [χ^2^_(DF=1)_ = 21.723, *p* < 0.001] as a main effect (Table 2 and Figure 2E). Experimental units with available space had faster germination than units containing *F. rubra*.

### Interaction Effects of Exposure Length, Disturbance, and Space Occupancy

**FIGURE 3:**
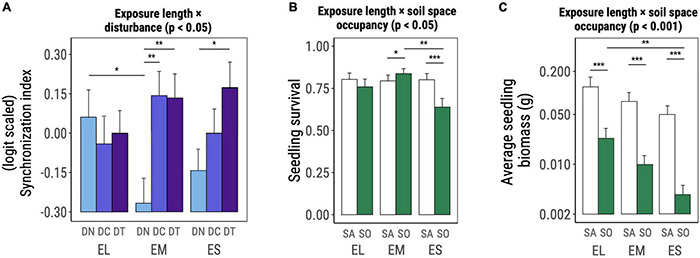
Significant interactive effects of exposure-length group × disturbance **(A)** and exposure-length group × space occupancy **(B,C)** on early life performance of the studied invasive plant species as determined based on Wald χ^2^ tests on (generalized) linear mixed effects models (significance level noted on top of the plot). Graphs show marginal means and standard errors estimated by the corresponding model. Horizontal bars denote significant differences among levels of each factor within levels of the other factor involved in the interaction and were assessed based on *post hoc* comparisons on marginal estimated means. Abbreviations and color codes: EL, exposure long species; EM, exposure medium species; ES, exposure short species; light blue DN: no disturbance; blue DC: disturbance compaction; purple DT: disturbance tilling; white SA: space available; green SO: space occupied. Significance levels: *p ≤ 0.05; **p ≤ 0.01; ***p ≤ 0.001.

The interaction between exposure-length group and disturbance significantly affected the synchronization of germination [χ^2^_(DF = 4)_ = 9.778, *p* = 0.044, Table 2 and Figure 3A]. In absence of disturbance, EL species had a significantly more synchronized germination when compared to EM species (*t*-ratio_EL–EM_ = 2.375 with *p* = 0.047, Figure 3A) but did not significantly differ from ES species. The application of a disturbance treatment drastically increased the germination synchronization of EM species (*t*-ratio_DN–DC_ = −3.146 with *p* = 0.005, *t*-ratio_DN–DT_ = −3.074 with *p* = 0.006, Figure 3A). For ES species, only the tilling treatment significantly increased the germination synchronization (*t*-ratio_DN–DT_ = −2.499 with *p* = 0.034, Figure 3A).

Seedling survival was significantly affected by the interaction between exposure-length group and space occupancy [χ^2^_(DF = 2)_ = 7.947, *p* = 0.019, Table 2 and Figure 3B]. EM species benefited from the presence of *F. rubra*, as shown by the higher survival in experimental units with occupied space (*t*-ratio_SA–SO_ = −2.119 with *p* = 0.035, Figure 3B). ES species were instead hampered by the grass, showing significantly lower survival in the occupied space treatment (*t*-ratio_SA–SO_ = 4.782 with *p* < 0.001, Figure 3B). EM species in presence of *F. rubra* had a significantly higher survival than ES species under the same treatment (*t*-ratio_EM–ES_ = 3.462 with *p* = 0.002, Figure 3B).

Average seedling biomass per experimental unit was also shaped by the interaction between exposure length and space occupancy [χ^2^_(DF=2)_ = 16.279, *p* < 0.001, Table 2 and Figure 3C]. All exposure-length groups had smaller seedlings when the space was occupied by *F. rubra*, whereas ES plants were the most strongly impacted in this sense (EL: *t*-ratio_SA–SO_ = 10.358 with *p* < 0.001, EM: *t*-ratio_SA–SO_ = 14.525 with *p* < 0.001, ES: *t*-ratio_SA–SO_ = 15.906 at with *p* < 0.001, Figure 3C). In absence of *F. rubra* the three exposure-length groups did not significantly differ in terms of seedling biomass; when the space was occupied, EL species outperformed ES seedlings (*t*-ratio_EL–ES_ = 3.481 with *p* = 0.015, Figure 3C).

None of the investigated early life performance traits were significantly shaped by the three-way interaction exposure length × disturbance × space occupancy.

## Discussion

Following the NPI hypothesis, we subdivided a set of invasive plant species into groups of different assumed exposure lengths to agropastoralism. We then investigated their early-life performance responses to simulated management practices, hypothesizing that plants with longer histories of exposure to agropastoral management would benefit more from the associated disturbances than species presumably lacking such co-evolution (and therefore pre-adaptation to disturbance). Our results provide no support for this hypothesis but rather support that the early-life performance of any invasive species is generally fostered by moderate soil disturbance (Catford et al., 2012). The lack of a clear differentiation between the species groups in response to disturbance raises questions regarding the assumption that Eurasian species possess a higher adaptation to agropastoralism. Our results support the idea that non-Eurasian species can too be adapted to such disturbance, as it is a practice developed independently in many regions worldwide before the arrival of European colonizers.

### Soil Disturbance and Available Soil Space Generally Promote Early-Life Performance in Plant Invaders

In accordance with the first hypothesis, all species benefited from disturbance and were impaired by space occupancy by *F. rubra*. Experimental units with disturbed soil had higher germination and seedling survival than untreated units (Figures 2C,D), while the presence of *F. rubra* slowed down the germination process and reduced seedling biomass in all groups, independent of the assumed length of exposure to agropastoralism (Figures 2E, 3C). These results add to a large body of literature on the facilitating effect of anthropogenic disturbance for biological invasions (Xiao et al., 2016; Meyer et al., 2021). Soil compaction and tilling increase contact between seeds and soil and create favorable germination microenvironments by buffering against mechanical damage as well as temperature and moisture variation (Burmeier et al., 2010; Limón and Peco, 2016; Eichberg and Donath, 2018). A limited availability of free space due to the presence of other species can be an obstacle to a successful early-life phase and it is one of the main drivers of community biotic resistance to invasion (Levine et al., 2004). The presence of grass individuals in the experimental units could hamper germination through several mechanisms, such as modification of the abiotic conditions experienced by the seed (e.g., light, moisture, or temperature regimes) (Weltzin et al., 2005), alteration of microbial community (Miller et al., 2019), and production of allelochemicals (Latif et al., 2017) (see section “Response to Space Occupancy Is Affected by Assumed Exposure Time to Agropastoralism” for further details).

### Assumed Length of Exposure to Agropastoralism Does Not Influence Response to Disturbance for Most Traits

Our results provided no support for the prediction that EL species (native to Western Asia, long exposure to agropastoralism) respond more positively to disturbance than EM plants (native to Europe, medium exposure to agropastoralism), and that EM plants respond more positively than ES species (native to America, short exposure to agropastoralism). The vast majority of early life performance responses to disturbance were independent from the assumed length of exposure to agropastoralism. Only germination synchronization responded more positively to disturbance in EM and ES species but had no effect on EL species, which have the presumed highest pre-adaptation to disturbance (Figure 3A).

These findings contradict the assumption that Eurasian species have a superior ability to cope with agropastoral disturbance due to several millennia of co-evolution with such practice. This is in line with other studies: a large-scale manipulative experiment, replicated across several study sites in the world, tested for an interactive effect of nutrient addiction (often connected to agropastoral management) and species’ introduction status and demonstrated that the leaf nutrient content of neither European invasive species nor of local native species was influenced by such interaction (Broadbent et al., 2020). Cultivation and animal husbandry are not exclusive to Western Asia and Europe but have surged independently through the millennia in different parts of the world. Researchers have identified eleven centers of plant domestication besides Western Asia, spanning from 8,000 BCE to 2,000 BCE (Purugganan and Fuller, 2009). In the Andes, for example, several edible species have been domesticated (e.g., *Solanum tuberosum* L., potato – Purugganan and Fuller, 2009) and native herbivores such as llama were raised in large numbers as source of sustenance and textile fibers (Flores Ochoa et al., 1994). All these practices must have exerted selection pressures on the local native plant species, leading to adaptation. Even though MacDougall et al. (2018) specify that the NPI hypothesis does not rest “on agricultural land use *per se*, but the specific combination of domesticated grazers, plants and management styles introduced simultaneously by Europeans following colonization,” there is no evidence supporting that European style agropastoralism should be profoundly different from practices found elsewhere. In addition, a standard definition of such practice is unrealistic as European settlers could have exported distinct sets of domesticated animals and plants, or even implement different cultivation styles according to the nationality of the exporters, thereby underlining the need for differentiation rather than generalization.

One further aspect to consider is that human landscape management is not the sole driver for adaptation to disturbances in relation to agropastoralism. Open herbaceous ecosystems are often inhabited by large native grazers which can also exert a selection pressure on local plants. In the Argentinian Patagonia for example, the long history of grazing by the native camelid guanaco (*Lama guanicoe* Müller) (Franklin, 1982) lead to the emergence of functional traits mediating grazing resistance in the local vegetation, when compared with a similar ecosystem that lacked large herbivores (Adler et al., 2004). Finally, as all of our seed material was collected in central Europe, we cannot exclude that the positive responses of ES species to agropastoral disturbance result from post-introduction adaptations. However, pre-adaptations are more likely to explain our results, given that ES species occur in cultivated areas and grasslands also in their native habitat (Table 1) and that disturbance acts as a strong environmental filter on non-adapted plant species (Huston and Smith, 1987). These pre-adaptations likely arose prior to the spread of agropastoralism with European settlers.

However, further experiments are required to validate these outcomes and conclusions. When monitoring plant response to agropastoral management, it is important to account for the multi-faceted character of disturbance, as determined by quality, intensity, frequency, duration, extent and timing (Zhang and Shea, 2012); which, if varied, can substantially shape the magnitude and direction of responses in plant performance. Mowing during flowering or fruiting periods, for example, is crucial for the management of invasive species, as it can affect plant resource allocation and growth form (Bartoš et al., 2011) but also dramatically reduce reproductive success, and consequently, population growth (Milakovic and Karrer, 2016; Nakahama et al., 2016). Responses to disturbance can also depend on plant life stage. Individuals in the germination, juvenile or maturity phase can be differently affected by environmental stimuli (Knappová et al., 2013; Florianová and Münzbergová, 2018). Jauni and Ramula (2017), for example, found out that for *Lupinus polyphyllus* Lindl. a moderate disturbance (comparable to our tilling treatment) increased germination but did not affect survival, while a stronger disturbance (removal of vegetation, litter and top 5 cm of soil) did not affect emergence but promoted seedling survival. Space occupancy can also affect plant life stages differently. In the case of *Impatiens parviflora* C., germination is impaired by high vegetation cover, which in turn does not affect survival later in life (Florianová and Münzbergová, 2018). Future experiments testing the NPI hypothesis should thus explicitly expand the variety of disturbances applied and consider also mature and reproductive life stages for an even higher number of species.

### Response to Space Occupancy Is Affected by Assumed Exposure Time to Agropastoralism

We expected that ES species would be more negatively affected by space occupancy than other groups, and that this would be particularly evident in the presence of disturbance. Even though we did not find support for the latter prediction, our results for seedling survival and final biomass support the former one. In fact, survival in the three exposure-length groups was differently affected by the presence of *F. rubra*, which had a neutral effect on EL species, a positive one on EM species and negative one on ES species (Figure 3B). In terms of final seedling biomass, all three exposure-length groups presented smaller seedlings when the space was occupied, with EL species being the least affected and ES species the most affected.

For survival, the identity of the species occupying the space has unlikely played a role, as *F. rubra* is widespread and native to the temperate areas of Eurasia and America. However, we used a cultivar of the species (Dipper) originating from Germany (OECD, 2021). Such cultivar, although not present in nature, has been developed using European populations of *F. rubra*, which can be found together with EM species in various phytosociological classes (Klotz et al., 2021) and therefore shares a particularly long co-evolutionary history with species belonging to this group. The facilitating effect of co-evolved species is assumed to have a great relevance in the context of biological invasions, during which the presence of another non-native species can benefit the establishment and proliferation of others (Simberloff and Von Holle, 1999) through reduction of competition from native plants (Flory and Bauer, 2014), alterations to the soil microbiome or other soil characteristics (Zhang et al., 2020) and allelopathy (Thiébaut et al., 2019). The same may hold for co-evolved species varieties or ecotypes. In a study by Lipińska et al. (2013), allelopathic effects of European cultivars of *F. rubra* (“Dipper” not included) were shown for grass species native to Europe. They observed a mixed outcome of species interactions (positive/negative) between European cultivars of *F. rubra* and the target European grass species investigated. One would not expect systematic variation in the susceptibility of plant species to the allelopathic effects of “Dipper” (in case this cultivar produces allelochemicals) among different exposure-length groups because even within one distribution range there is plenty of variation in the allelopathic potential of cultivars and the effects of particular cultivars on different target species.

The interactive effect of exposure-length group and space occupancy on seedling biomass (Figure 3C) could be mediated by a positive correlation between germination speed and seedling size (Grman and Suding, 2010), as individuals from early germinating species (EL) may have had the chance to establish and occupy space before the *F. rubra* cover was excessively dense. In temperate habitats where plant development is constrained during the cold season, an early germination could offer an invasive species the chance to exploit local resources and therefore be detrimental for the establishment and diversity of later-germinating native species (Grman and Suding, 2010). Regarding seedling biomass, it could be argued that large-seeded species would have an advantage as they would have a higher recruitment and generate larger seedlings (Moles et al., 2004). However, this advantage diminishes significantly in ecosystems with sparse canopy such as grasslands (Bruun and Ten Brink, 2008) and it is rather unlikely to apply in the present case as all of our groups possessed both large- and small-seeded species.

## Conclusion

By testing for the combined effects of space occupancy and disturbance on the performance of introduced plants we found no evidence suggesting that EL and EM species are more adapted to agropastoralism than ES species. In contrast, the latter can cope with the disturbances in early life phases just as well as Eurasian species. However, even though our experimental setup comprised a representative sample of species for the three exposure-length groups that allowed for generalization of the combined effects of soil surface disturbance and space occupancy, a different/larger set of species with a more balanced phylogenetic relatedness may have yielded contrasting results. Further studies are required to expand our findings and evaluate whether EL, EM, and ES species react differently to agropastoral disturbance and space occupancy in later life stages. The complex interplay of pre-adaptation to disturbance, the quality/frequency/intensity of disturbance and the species composition at site is a determinant of invasion success that must be addressed in future studies of this kind. These multifaceted interactions should be assessed employing manipulative experiments in the field, that address plant performance across the entire life cycle (including fitness) and that simultaneously allow modeling population growth rates.

## Data Availability Statement

The raw data supporting the conclusions of this article will be made available by the authors, without undue reservation.

## Author Contributions

AE acquired funding and contributed to the final version. GB, AE, and KS conceptualized the study. GB implemented the experiment and collected the data. GB and KS performed the statistical analysis and wrote and revised the manuscript. All authors approved the submitted version.

## Conflict of Interest

The authors declare that the research was conducted in the absence of any commercial or financial relationships that could be construed as a potential conflict of interest.

## Publisher’s Note

All claims expressed in this article are solely those of the authors and do not necessarily represent those of their affiliated organizations, or those of the publisher, the editors and the reviewers. Any product that may be evaluated in this article, or claim that may be made by its manufacturer, is not guaranteed or endorsed by the publisher.
