# Supplementary material for: No Support for the Neolithic Plant Invasion Hypothesis: Invasive Species From Eurasia Do Not Perform Better Under Agropastoral Disturbance in Early Life Stages Than Invaders From Other Continents
Source: Front Plant Sci. 2022 Feb 11;13:801750. doi: 10.3389/fpls.2022.801750 (PMC8874271; doi:10.3389/fpls.2022.801750)
Supplement: Supplementary file 1 [file Data_Sheet_1.docx]

Supplementary Material 1

Figure 1 (A) Soil occupancy treatment: planting position of 11 individuals of *Festuca rubra* (green rectangles) on soil surface. (B) Soil compaction treatment: placement of hoof-shaped trampling device on soil surface. Black large circle represents the external border of the experimental unit.
