# Supplementary material for: No Support for the Neolithic Plant Invasion Hypothesis: Invasive Species From Eurasia Do Not Perform Better Under Agropastoral Disturbance in Early Life Stages Than Invaders From Other Continents
Source: Front Plant Sci. 2022 Feb 11;13:801750. doi: 10.3389/fpls.2022.801750 (PMC8874271; doi:10.3389/fpls.2022.801750)
Supplement: Supplementary file 2 [file Data_Sheet_2.docx]

Supplementary Material 2

# Testing for significant difference in seed mass between exposure-length groups

**# LIBRARIES**

library(car)

**# READ IN DATA**

#Dataframe contains species identity, exposure-length group affiliation and average seed mass obtained from BiolFlor (Klotz et al., 2021) or the Kew Botanical Gardens Seed Information Database (Royal Botanic Gardens Kew, 2021)

data_w <- read.csv("Weights species WP1.csv", h=T, stringsAsFactors = T)

**# KRUSKAL-WALLIS RANK SUM TEST**

# Assumptions for Anova analysis were not met (Shapiro-Wilk normality test on resuduals, p = 2.291e-08)

kruskal.test(Weight ~ Group, data = data_w)

# List of "best" fit models in which the species ranef is weighted for phylogenetic relatedness

# Adjustment included:

# varying among appropriate link functions

# trying different appropriate response data transformations (log, sqrt, logit, z)

# adding appropriate dispersion formulas

# adding appropriate zero-inflation formulas

# none of the models resulted in unproblematic residual diagnostic plots

**# LIBRARIES**

devtools::install_github("glmmTMB/glmmTMB/glmmTMB")

devtools::install_github("wzmli/phyloglmm/pkg")

install.packages(c("phyloglmm","glmmTMB","lme4","DHARMa","car","emmeans","ggplot2","ggsignif","ggpubr","insight","ape","Matrix","dplyr"))

library(phyloglmm)

library(glmmTMB)

library(lme4)

library(DHARMa)

library(car)

library(emmeans)

library(ggplot2)

library(ggsignif)

library(ggpubr)

library(insight)

library(ape)

library(Matrix)

library(dplyr)

**# PREPARING PHYLOGENETIC MATRIX**

**# Function** (Li and Bolker, 2021)

phylo.to.Z <- function(r,stand=FALSE){

ntip <- length(r$tip.label)

Zid <- Matrix(0.0,ncol=length(r$edge.length),nrow=ntip)

nodes <- (ntip+1):max(r$edge)

root <- nodes[!(nodes %in% r$edge[,2])]

for (i in 1:ntip){

cn <- i

while (cn != root){

ce <- which(r$edge[,2]==cn)

Zid[i,ce] <- 1

cn <- r$edge[ce,1]

}

}

V <- vcv(r)

# V <- V/max(V)

sig <- exp(as.numeric(determinant(V)["modulus"])/ntip)

# sig <- det(V)^(1/ntip)

Z <- t(sqrt(r$edge.length) * t(Zid))

if(stand){Z <- t(sqrt(r$edge.length/sig) * t(Zid))}

rownames(Z) <- r$tip.label

colnames(Z) <- 1:length(r$edge.length)

return(Z)

}

**# Read in tree**

phylo_my <- read.nexus("tree_ok.nex")

**#Create tree matrix object**

system.time(phyloZ_my <- phylo.to.Z(phylo_my))

**# READ IN DATA**

data <- read.csv("WP1_NPIH_data_2.csv",dec=".", header=TRUE,stringsAsFactors = T)

data <- (data %>% mutate(Species = factor(Species,levels = phyloZ_my@Dimnames[[1]])))

**# Calculate germination success**

data$G_succ<-cbind(data$G_max,data$No_G_max)

**# Calculate survival**

data$survived<-data$G_final

data$not_survived<-data$G_max-data$G_final

data$survival <- cbind(data$survived,data$not_survived)

**# MODELS**

**# Germination success**

m_G_p <- phyloglmm:::phylo_glmmTMB(G_succ~Group*Competitor*Disturbance+(1|Species)

, data=data

, family="betabinomial"

, phyloZ=phyloZ_my

, contrasts=list(Disturbance="contr.sum",Group="contr.sum",Competitor="contr.sum")

, phylonm = "Species"

)

sim_G_p <- simulateResiduals(m_G_p, n=2000)

plot(sim_G_p)

**# Germination speed**

m_MR_p <- phyloglmm:::phylo_glmmTMB(log(MR)~Competitor*Group*Disturbance+(1|Species)

, contrasts=list(Disturbance="contr.sum",Group="contr.sum",Competitor="contr.sum")

, family="gaussian"

, phyloZ=phyloZ_my

, data= data

, phylonm = "Species"

, REML = FALSE

)

sim_MR_p <- simulateResiduals(m_MR_p, n=2000)

plot(sim_MR_p)

**# Synchrony**

data$Z_logit <- logit(data$Z, percents=max(data$Z, na.rm = TRUE) > 1)

data = data %>% group_by(Species) %>% mutate(Z_ls = scale(Z_logit, center = T))

data$Z_ls <- as.numeric(data$Z_ls)

m_Z_p <- phyloglmm:::phylo_glmmTMB(Z_ls~Competitor*Group*Disturbance+(1|Species)

, contrasts=list(Disturbance="contr.sum",Group="contr.sum",Competitor="contr.sum")

, family=gaussian()

, phyloZ=phyloZ_my

, data= data

, phylonm = "Species"

, REML = FALSE

)

sim_Z_p <- simulateResiduals(m_Z_p, n=2000)

plot(sim_Z_p)

**# Seedling survival**

m_S <- phyloglmm:::phylo_glmmTMB(survival~Competitor*Group*Disturbance+(1|Species)

, contrasts=list(Disturbance="contr.sum",Group="contr.sum",Competitor="contr.sum")

, family=betabinomial

, phyloZ=phyloZ_my

, data= data

, phylonm = "Species"

)

sim_S <- simulateResiduals(m_S, n=2000)

plot(sim_S)

**# Seedling biomass**

m_W_p <-phyloglmm:::phylo_glmmTMB(log(Avg_ind)~Competitor*Group*Disturbance+(1|Species)

, contrasts=list(Disturbance="contr.sum",Group="contr.sum",Competitor="contr.sum")

, family="gaussian"

, phyloZ=phyloZ_my

, data= data

, phylonm = "Species"

, REML = FALSE

)

sim_W_p <- simulateResiduals(m_W_p, n=2000)

plot(sim_W_p)

# References

Klotz, S., Kühn, I., and Durka, W. (2021). BIOLFLOR - A database on biological-ecological characteristics of vascular plants in Germany. *UFZ Cent. Environ. Res. Leipzig-Halle*.

Li, M., and Bolker, B. (2021). phyloglmm: Machinery for phylogenetic GLMMs.

Royal Botanic Gardens Kew (2021). Seed Information Database (SID). Available at: http://data.kew.org/sid/.
